# A Scalable Framework for Telehealth: The Mayo Clinic Center for Connected Care Response to the COVID-19 Pandemic

**DOI:** 10.1089/tmr.2020.0032

**Published:** 2021-02-24

**Authors:** Tufia C. Haddad, Rebecca N. Blegen, Julie E. Prigge, Debra L. Cox, Greg S. Anthony, Michelle A. Leak, Dwight D. Channer, Page Y. Underwood, Ryan D. Williams, Rhapsody D. Hofschulte, Laura A. Christopherson, Jordan D. Coffey, Sarvam P. TerKonda, James A. Yiannias, Brian A. Costello, Christopher S. Russi, Christopher E. Colby, Steve R. Ommen, Bart M. Demaerschalk

**Affiliations:** ^1^ Mayo Clinic, Rochester, Minnesota, USA.; ^2^ Mayo Clinic, Jacksonville, Florida, USA.; ^3^ Mayo Clinic, Scottsdale, Arizona, USA.

**Keywords:** COVID-19, remote patient monitoring, telehealth, telemedicine, virtual care

## Abstract

**Background:** The Mayo Clinic Center for Connected Care has an established organizational framework for telehealth care delivery. It provides patients, consumers, care teams, and referring providers access to clinical knowledge through technologies and integrated practice models. Central to the framework are teams that support product management and operational functions. They work together across the asynchronous, synchronous video telemedicine, remote patient monitoring (RPM), and mobile core service lines.

**Methods:** The organizational framework of the Center for Connected Care and Mayo Clinic telehealth response to the COVID-19 pandemic is described. Barriers to telehealth delivery that were addressed by the public health emergency are also reported. This report was deemed exempt from full review by the Mayo Clinic IRB.

**Results:** After declaration of the COVID-19 pandemic, there was rapid growth in established telehealth offerings, including patient online services account creation, secure messaging, inpatient eConsults, express care online utilization, and video visits to home. Census for the RPM program for patients with chronic conditions remained stable; however, its framework was rapidly adapted to develop and implement a COVID-19 RPM service. In addition to this, other new telehealth and virtual care services were created to support the unique needs of patients with COVID-19 symptoms or disease and the health care workforce, including a digital COVID-19 self-assessment tool and video telemedicine solutions for ambulances, emergency departments, intensive care units, and designated medical–surgical units.

**Conclusion:** Rapid growth, adoption, and sustainability of telehealth services through the COVID-19 pandemic were made possible by a scalable framework for telehealth and alignment of regulatory and reimbursement models.

## Overview

The Center for Connected Care is the home to Mayo Clinic's telehealth experience and care delivery. It provides patients, consumers, care teams, and referring providers access to Mayo Clinic's expertise and knowledge through technologies and integrated practice models at the right time and place to best meet their needs. These shared services facilitate optimal care for patients at a distance, enable the practice to improve access for complex patients who require in-person diagnosis and treatment, and aim to decrease the overall cost of care.

**FIG. 1.**
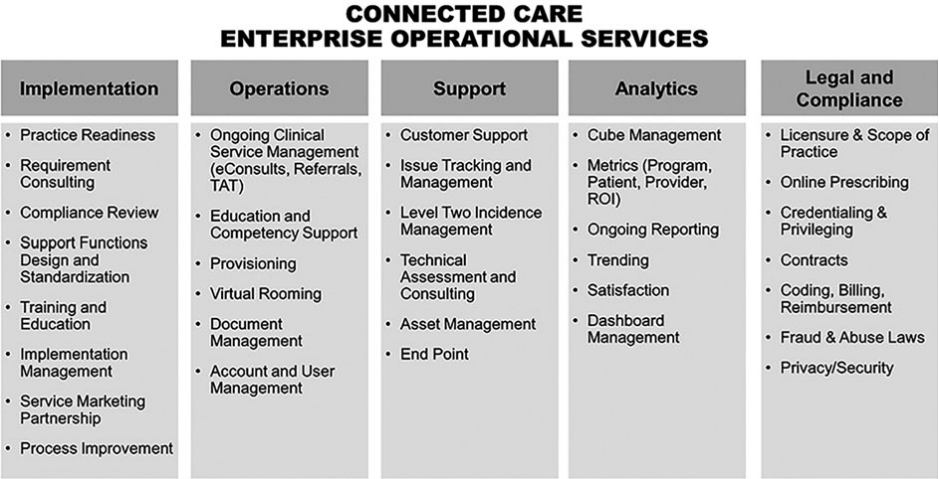
Essential elements of operational infrastructure.

Since 2012, the Center for Connected Care experience has demonstrated that a successful organizational framework for telehealth does not solely focus on the technology, but includes both product management and operational functions that work closely together to provide support across core product lines. The product management domain has product managers, information technology, and user design resources that support the full spectrum of telehealth products for web and mobile platforms, as well as their electronic health record (EHR) integration. The product teams are responsible for ensuring that the strategic direction, planning, execution, and delivery of products and services support the organization's overall telehealth strategy and goals. Operations include five key pillars of activities critical to support digital care solutions within the practice,^1^ including implementation training and resources, operational service management, customer and technical support, analytics, and legal and compliance. See Figure 1 for additional details related to each.

Core product lines include asynchronous, synchronous video telemedicine, remote patient monitoring (RPM), and mobile. A medical director and administrative partner provide leadership for each of the core product lines with site leadership at each of the Mayo Clinic campuses in Minnesota, Arizona, Florida, and the community-based Mayo Clinic Health System (MCHS) practice spanning three states in the Midwest.

**FIG. 2.**
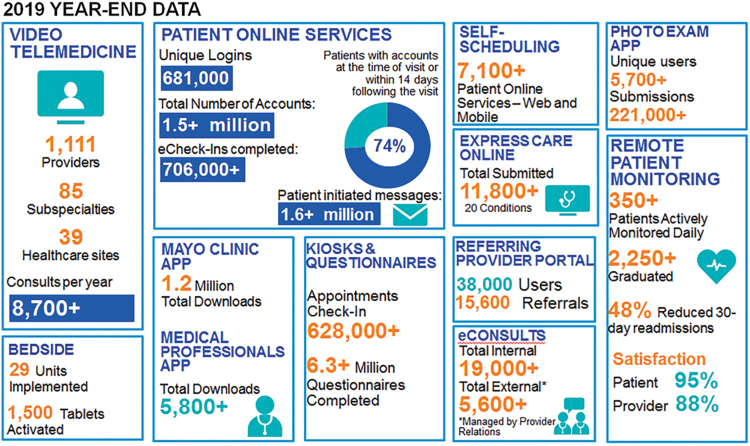
The Center for Connected Care 2019 year-end data.

In 2019, Connected Care supported over 1.5 million patient online service (POS) accounts, over 1.6 million patient-initiated secure messages, 8700 video consultations, 2250 RPM patients, and 1.2 million downloads of the Mayo Clinic mobile app. A summary of this and additional data related to telehealth and virtual care activities in 2019 are provided in Figure 2. This technical, operational, and administrative infrastructure positioned Mayo Clinic well to respond to the COVID-19 pandemic.

### Asynchronous services

Mayo Clinic POS (patient portal) was established in 2010 as a service offering to support bidirectional communication between patients and providers via *secure messaging*. Growth of secure messaging through the portal was fueled by the emergence of the EHR, its improved functionality, and the U.S. federal meaningful-use program. At Mayo Clinic, more than 5 million secure messages were exchanged via the patient portal between February 18, 2010, and December 31, 2017.

Several additional asynchronous services provide patients with access to their care teams for specialty consultations that do not require in-person evaluation. *eConsults* are asynchronous, electronic consultations between Mayo Clinic providers established to support specific focused clinical questions regarding patients in an outpatient or inpatient setting. *Express Care Online* is a digital service in which a patient completes a symptom-specific questionnaire through the portal, the results of which are reviewed and triaged within an hour by an advanced practice provider, and care recommendations are provided back via secure messaging. Up to 20 minor subacute symptoms/conditions (such as cystitis, ear pain, and pink eye) are supported.

### Video telemedicine

Video telemedicine enables virtual access to clinical expertise for consultation, guidance, and follow-up care. Video services span clinical settings and can be delivered to patients at home, in a facility, or during transport. In 2015, significant steps were taken to develop secure, reliable, and scalable infrastructure to support video telemedicine at Mayo Clinic. A foundation for long-term scalability and growth was generated by centralizing core operations and deploying robust technology solutions to support acute and outpatient needs.

Outpatient telemedicine is offered to patients at a remote health care facility or directly to the patients at home. Outpatient video appointments to another care facility provide patients access to specialty care at their local health care facility, reducing travel time and costs for both patients and providers. Mayo Clinic offers video consults to over 33 community-based Mayo Clinic Health System practices by 35 specialties. Video appointments to home enable providers at a Mayo Clinic facility to connect with the patients in their home, saving patients the time and expense of returning to a Mayo Clinic site for follow-up care. Before the COVID-19 pandemic, video appointments to the patient at home were primarily utilized for routine follow-up appointments by select surgical and specialty practices and comprised <1% of overall visit volumes.

Acute care telemedicine provides specialty consultation and remote care guidance for medical and surgical emergencies through live video communication. Acute telemedicine services such as tele-emergency medicine, telestroke, and teleneonatology reduce patient transfers and improve patient outcomes.

### Remote patient monitoring

In 2015, the RPM program was established to ensure standardized and scalable services with centralized program management, clear governance and oversight, standardized clinical practice and reporting, and partner and vendor management. Two separate care models and technology platforms provide a spectrum of care offerings.

The high-intensity RPM model involves a patient at home who receives a technology package that comprised a tablet with cellular service and Bluetooth-enabled devices (blood pressure cuff and monitor, pulse oximeter, thermometer, and scale). The tablet notifies patients to perform vital sign measurements and complete condition-specific symptom assessments at least once daily. The tablet can also facilitate the delivery of education and video-enabled assessments. Patient-reported data, passively collected or manually entered, trigger alerts based on predetermined parameters. Key to this RPM model is a centralized team of registered nurse care coordinators (RPM RNs) who provide daily monitoring and education, respond to alerts and missed tasks, leverage standardized decision trees and protocols for interventions, and escalate to the appropriate managing clinician or department as necessary. This RPM model was established to manage patients paneled in primary care with complex and/or suboptimally controlled chronic conditions who have high resource utilization, risk for readmission, and/or a prolonged hospital stay. Average program length was ∼90 days. The RPM care model was implemented in the Mayo Clinic Health System sites from 2016 to 2018. Efforts to further expand across all Mayo sites and to the specialty practice were initiated in 2019, and over 3000 patients had been served on the program through 2019 at an average daily census of 350.

Low-Intensity Monitoring: condition-specific, Interactive Care Plans (ICPs) were developed for patients with low acuity chronic disease or a limited medical event (surgery, pregnancy, acute infections, etc.). This RPM model is made available on patient-owned smart devices through the Mayo Clinic mobile app, and each ICP provides patients with actionable guidance aimed to facilitate self-management. Patients are notified via their mobile device to self-report biometric data and symptoms associated with their condition, view educational content, and track their medications. All data are seamlessly EHR-integrated and viewable to the patient's care team. If the data are outside predetermined parameters, 1 of 2 pathways for escalation will occur: for minor deviations, just-in-time education is delivered by mobile to foster self-management, and for moderate-to-severe deviations signaling adverse health trends, an alert is sent via the EHR to the patient's care team or the patient is instructed to contact emergency services. Nine ICPs have been codeveloped with Epic software engineers, the first of which was implemented into the Heart Failure Clinic practice in November 2019. Other ICP implementations were delayed due to COVID-19 response, but subsequently resumed in August 2020.

### Mobile health

Utilization on smartphones, mobile apps, and wearables has experienced explosive growth over the past decade. The mobile platform was established to provide common technical structure and oversight to available and future Mayo Clinic apps. Mobile app development efforts are primarily focused on leveraging current and emerging mobile technologies to extend health care services beyond traditional care settings and to expand access to Mayo Clinic information for patients, medical professionals, and health seekers. Mayo Clinic apps are powered by trusted health content developed collaboratively by more than 4700 physicians and scientists, and they are integrated across the asynchronous, video telemedicine, and RPM product lines.

## Overcoming Barriers to Telehealth Adoption in Response to the COVID-19 Pandemic

Despite robust technical, operational, and administrative infrastructure to support telehealth and virtual care, numerous factors limited widespread adoption of products and services by providers and patients. The requirement of medical licensure in the state in which the patient resides prevented many health care providers from extending ongoing care across state lines. There were also limited means for reimbursement by the Center for Medicare & Medicaid Services (CMS) or commercial insurers for telehealth services. Even with established self-pay models, which have been shown to save patients money by reducing time away from work and negating the need to travel for care,^2^ video and/or telephone visits comprised <1% of all ambulatory visits in 2019. The lack of familiarity with the technical requirements to facilitate virtual care and concerns for technical failures further limited provider and patient adoption.

Between March and May 2020, to reduce health care worker, patient, and caregiver exposure to COVID-19 and limit utilization of personal protective equipment (PPE) required for in-person care, there was an unparalleled expansion and utilization of digital health solutions by providers and patients. To enable rapid growth, several legal and regulatory barriers to telehealth and virtual care adoption were removed.

### Licensure barriers

In 2020, licensure of health care professionals falls within the authority of each state, requiring many individual states' licenses for multistate practices. During a telehealth encounter, the provider must adhere to the state licensing rules and regulations for both the state where the patient is located and the state where the provider is located. The licensing process is time-consuming and expensive, and varying continuing medical education requirements add administrative burden to maintain multiple state licenses.

In response to the COVID-19 pandemic, the federal and state governments took emergency action to temporarily relax or remove the licensing barriers and expanded scopes of practice for physicians and many other licensed health care professionals.^3^ The Drug Enforcement Administration also adopted policies to allow registered practitioners to prescribe controlled substances without having to interact in person with the patients.^4^ By removing these barriers, telehealth services could be readily deployed across state lines to ensure that hospitals and clinics had the capacity to manage surges and maintain access for those requiring advanced diagnostics and treatment on-site.

### Billing and reimbursement barriers

Historically, both Medicare and commercial payer access to and reimbursement for telehealth services have been restrictive. On March 27, 2020, the U.S. Congress approved the Coronavirus Aid, Relief, and Economic Security (CARES) Act, which included several key telehealth provisions.^5^ The CARES legislation codified the executive action taken by the Secretary of Health & Human Services to declare a federal state of public health emergency (PHE)—as allowed under the Public Health Services Act. This PHE declaration was subsequently renewed in April, July, and October of 2020 for consecutive 90-day periods.^6^ CARES expanded Medicare coverage by removing the requirement that a preexisting patient/provider relationship must be established before receiving telehealth services. The CMS issued guidance to expand telehealth in the Medicare program under the CARES Act and the Section 1135 waiver authority.^7^ These waivers, authorized under the Public Health Services and Social Security Acts, offer states new flexibilities in combating the outbreak, including offering or expanding telehealth to Medicaid beneficiaries in their states.

While many of the changes enacted at the federal level impacted reimbursement and coverage by CMS, commercial insurers updated existing polices or created new temporary telehealth policies for coverage and reimbursement of similar services. This allowed patients, regardless of insurance coverage, access to medical care without having to be physically present with their provider. Furthermore, on March 30, 2020, the CMS expanded telehealth services and RPM capabilities, allowing providers to supervise from an off-site setting virtually through a real-time audio/visual connection, and permitted out-of-state practitioners to provide telehealth services in other states.^8^

### Connectivity barriers

Telehealth services require patients to utilize a computer with a broadband internet connection and/or a cellular-enabled smartphone or tablet. Ownership of these connected devices and services is steadily increasing, but differences in connectivity are recognized by household owner's age, location, race/ethnicity, and income.^9^ The CARES Act further allocated funds for health care organizations to implement connected telehealth infrastructure for patient care through the PHE. Increased funding included a $200 million grant program administered by the Federal Communications Commission (FCC) to support organizational response to the COVID-19 emergency by providing telehealth services through connected devices. FCC awarded organizations up to a maximum of $1 million to cover the costs of connected care devices.^10^ These actions removed previous geographic restrictions that limited coverage to rural areas and certain health care facilities, not including home; thus, patients could receive telehealth services regardless of their locations.

## Growth of Existing Telemedicine Services in Response to the COVID-19 Pandemic

Health care systems had a unique, time-sensitive opportunity to capitalize on the easing of telehealth barriers and accelerate adoption of digital tools. Those already invested in telemedicine were well positioned to test scalability of digital health care. When the World Health Organization declared COVID-19 a pandemic on March 11, 2020, and institutional leadership deferred all elective and nonemergent care, the Center for Connected Care anticipated the increased demand for digital care solutions by rapidly preparing to expand existing services and developing new products and services aimed to preserve access to health care and support patient and staff safety needs across the continuum of care.

### Collaboration with enterprise Hospital Incident Command System

**FIG. 3.**
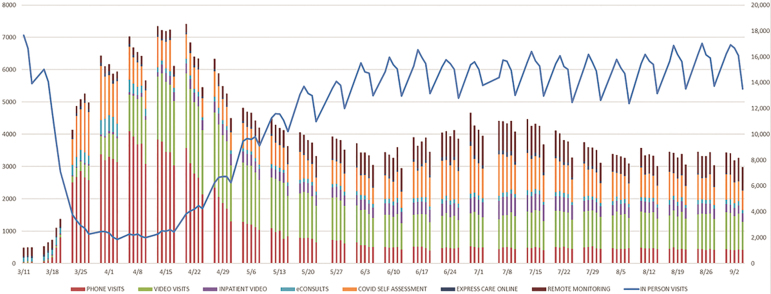
Growth of telehealth and virtual care services during the COVID-19 pandemic.

Mayo Clinic Hospital Incident Command System (HICS) is a leadership team activated when an emergency situation surpasses or is anticipated to surpass normal operating capabilities of the institution or departments. At the onset of the pandemic and to prepare for the wave of COVID-19 illness at each site, tight coordination between site HICS and Connected Care leadership teams was required to develop comprehensive plans that addressed several system, provider, and patient needs. The site surge plan's primary components required redirecting resources (staff, physical space, and equipment) to the frontline services. Connected Care deployed technology from its acute care telemedicine product portfolio and developed new Express Care Online and RPM services to address limited PPE supplies, foster communication between patients and care team members, and reduce staff exposure to patients who were suspected or confirmed to have COVID-19. Simultaneously, a focused effort across all clinical departments was to transition most ambulatory appointments to telemedicine (video or phone) consultations to provide care for new and established patients. A rapid response strategy by the Connected Care leadership allowed for immediate telemedicine service implementations or product deployments to meet the rapidly changing needs of the clinical practice. Weekly changes in the volume of specific telehealth and virtual care services, relative to in-person encounters, from March through August 2020, are highlighted in Figure 3.

### Growth in POS accounts, logins, and secure messaging

A significant increase in newly established POS accounts was observed in response to the COVID-19 pandemic as restrictions were imposed on traditional face-to-face care and patients desired to limit their exposure to the novel coronavirus. Technical infrastructure and personnel support, as well as letters explaining POS benefits during the pandemic, was provided by Connected Care to enable new account creation. In June and July 2020, the monthly new POS account creation jumped to 28,634 and 34,493, respectively, from a 2019 monthly average of 17,000.

Similarly, the number of POS logins trended upward throughout 2020, likely a result of other key telemedicine services offered through the portal, as POS serve as the mechanism to enable secure messaging, view COVID test results immediately, launch video telemedicine visits, complete Express Care Online self-assessments, and support the low-intensity RPM platform.

Secure messaging became an even more valuable tool for communicating COVID-19-related issues, including new symptoms, test results, and appointment rescheduling. In fact, in March 2020 with the PHE declaration and many states issuing shelter-in-place orders, there was a spike in the number of total secure messages. At that time, many in-person appointments and surgeries/procedures were being converted to virtual appointments or deferred. Interestingly, both secure messages and POS login trends were stable or declined during April, but then significantly increased again in June and July 2020, which tracked with the U.S. summer surge in COVID-19 cases as well as the phased reopening of the states and economy.

### Changes in outpatient and inpatient eConsult services

Before the pandemic, ambulatory eConsult services were mature across all specialty practices to support internal consult requests from within the organization and external consult requests from non-Mayo-owned, affiliated practices within the Mayo Clinic Care Network. Inpatient eConsult services, however, were limited with only two use cases being supported by prior successful pilots involving infectious disease and dermatology. During the initial months of the pandemic, there was a decline in eConsult volume, consistent with an overall reduction in practice and new patient volumes (Fig. 3). Whereas inpatient eConsults were made available to all hospital specialties and locations, and this service was broadly utilized.

### Utilization of Express Care Online

Cyclical usage of Express Care Online has been observed with higher utilization during the winter months and lower rates during the summer, averaging between 800 and 1200 eVisits per month. A spike in utilization occurred in March 2020 as the pandemic started as patients could immediately utilize Express Care Online to seek care while other telehealth options were developed or ramped up (Fig. 3).

### Growth in virtual care visits to home

As Mayo Clinic made the shift to reduce in-person appointments in March 2020, patients and care teams embraced virtual appointments by phone or video as an alternative care delivery model (Fig. 3). In April 2020, virtual appointments peaked at 5884 visits per day, and made up close to 70% of all outpatient appointments. During the interval from March 11 to April 20, 2020, specifically, the organization witnessed a 10,880% increase in video appointments to home, and a 13,650% increase in telephone appointments to home.^11^ Although virtual visit appointment volumes decreased as in-person visits resumed, the daily demand for these visits stabilized by June 2020 at ∼1600 per day (10% of total outpatient practice volumes). As clinical teams and the Center for Connected Care gained experience with virtual appointments, efforts were continually undertaken to reduce patient cancelations and “no show” events. These improvement efforts focused around the domains of patient communications, technical support, and process simplification. In addition, efforts are ongoing to better integrate virtual rooming processes directly into clinical workflows—moving from a centralized process by Connected Care staff to a decentralized one in clinical departments.

### RPM for chronic disease management

The majority of patients receiving high-intensity RPM had a primary diagnosis of congestive heart failure, COPD, or hypertension managed on the program. With the pandemic spreading across the United States in the early months of 2020, further growth and implementation of the program across new Mayo sites and specialty practices were paused. The daily census was intentionally decreased to ∼200 patients to prepare for growth and a potential surge of patients who tested positive for COVID-19, had at least one risk factor for severe disease, and did not require immediate hospitalization. Throughout the pandemic, this daily census for patients with chronic conditions (without COVID-19) has been maintained at 200, allowing vulnerable adults at risk for COVID-19 to have their conditions remotely managed outside of ambulatory clinic, ED, and hospital facilities. The RPM daily census over time is reported in Figure 3.

## Development and Diffusion of Novel Connected Care Services to Support Patients with COVID-19

### COVID-19 screening and diagnosis

An anonymous COVID-19 self-assessment tool was created to offer patients and Mayo Clinic employees a quick, self-service method to assess their symptoms and direct them to the appropriate next steps, such as quarantine and COVID-19 testing. This tool was made available on March 23, 2020, within the POS web-based portal and Mayo Clinic mobile app, as well as the Mayo Clinic Employee internet. The assessment tool is updated on a regular basis to reflect the latest recommendations from the published Infection Prevention and Control guidelines. Utilization of the COVID-19 self-assessment has grown with time, averaging 25,000 completed assessments per month (April to August 2020), as reported in Figure 3.

### Telemedicine support to deliver COVID-19 acute care in emergency medical services, emergency departments, intensive care units, and hospital wards

For patients with COVID-19 or suspected COVID-19 requiring acute care services, several video telemedicine solutions were deployed to support prehospital emergency medical services and all hospital emergency departments and intensive care units. These solutions helped minimize staff and patient exposures, played an instrumental role in conserving PPE, and kept patients connected with their family at home when visitation was limited. Based on hospitals' surge plan estimates, Connected Care was able to deploy over 800 audio–video telemedicine end-points across the enterprise, in Mayo Clinic ambulances, emergency departments, intensive care units, and in designated medical–surgical units.^12^

### Ambulatory and postdischarge COVID-19 management with telehealth and RPM services

At the onset of the pandemic, Mayo Clinic patients with a diagnosis of COVID-19, who were fully managed in the ambulatory setting or discharging from the hospital, stayed in contact with their care teams via POS secure messaging and video appointments. COVID-19 care teams (CCTs) were established in the Mayo Midwest (serving Rochester, MN, and MCHS), Arizona, and Florida, each comprising nurses and general internists or infectious disease specialists.^13^ These centralized CCTs were developed to ensure that the rapidly evolving best practices were adopted for COVID-19 disease management and hand-offs with regional emergency departments and COVID-19 hospital units were optimized. To deliver care at a broader scale, the HICS and hospital/outpatient practice leadership teams endorsed the development of a COVID-19 RPM program in partnership with the CCTs.

The existing RPM program framework supporting patients with chronic conditions was rapidly adapted to meet the unique needs of patients with acute COVID-19 and variable risk for severe illness. In addition, the program needed to support special patient populations not previously served by RPM, including those who were non-English speaking, pregnant, employees, and those who had prior transplant or active cancer. Vital sign parameters and symptom assessments for disease monitoring and alerts were established in collaboration with CCT physicians, as well as experts from hospital internal medicine and pulmonary/critical care. All adult patients (age ≥18 years) with COVID-19 were considered for RPM, with those at risk for severe illness being offered high-intensity monitoring, and those without risk factors offered low-intensity monitoring at the discretion of the CCT physicians. For high-intensity RPM, full vitals (blood pressure, heart rate, oxygen saturations, temperature, and weight) and symptom questionnaires were assessed twice daily, with the exception of immunosuppressed cancer and transplant patients who were assessed four times daily. For low-intensity monitoring, the same symptom questionnaires were utilized with a limited vitals' assessment (oxygen saturations, respiratory rate, and temperature) twice daily. The RPM RN care coordinators responded to all patient alerts and further escalated to CCT physicians when clinically appropriate.

The program was implemented across the Mayo Clinic enterprise, initially in the Midwest and then to Florida and Arizona sites from late March through mid-May 2020 with the aim to facilitate supportive care and disease monitoring to patients self-isolating at home (or elsewhere) during their recovery. From March 20 to August 20, 2020, the COVID-19 RPM program served 4651 unique patients across the Mayo Clinic sites, including 2507 (54%) patients with high-intensity monitoring, and 2144 (46%) with the low-intensity monitoring. The dynamic changes in the RPM daily census as regional surges occurred are reflected in Figure 3. Further details related to program development, implementation, patient utilization, and clinical outcomes will be reported in a separate article.

## Conclusion

The COVID-19 pandemic and shift toward digital health solutions have created a deep repository of cases from major medical institutions connecting with their patients. We share the Mayo Clinic Center for Connected Care telehealth product and operations framework with the aim to support and sustain newly formed programs at other health care systems that have emerged from the pandemic. It will be essential that as we move from crisis management roll out of services that we research the quality, reliability, and patient-centered factors that these models have provided. Preservation of changes to regulatory guidelines, connectivity infrastructure, and reimbursement models that made growth and adoption of services will be critical to long-term sustainability of telehealth. It will be through these collective efforts that we will obtain a better understanding of how to advance the science and practice of telehealth and virtual care delivery.

## Abbreviations Used

CARES: Coronavirus Aid, Relief, and Economic Security
CCT: COVID-19 care team
CMS: Center for Medicare & Medicaid Services
EHR: electronic health record
FCC: Federal Communications Commission
HICS: Hospital Incident Command System
ICP: Interactive Care Plans
PHE: public health emergency
POS: patient online services
PPE: personal protective equipment
RN: registered nurse care coordinators
RPM: remote patient monitoring
